# Correspondence on “An International Study Evaluating Elemental Analysis”

**DOI:** 10.1021/acscentsci.2c01484

**Published:** 2023-03-24

**Authors:** Richard J. C. Brown

**Affiliations:** National Physical Laboratory, Hampton Road, Teddington TW11 0LW, United Kingdom; University of Surrey, Guildford GU2 7XH, United Kingdom

## Abstract

This Correspondence provides a brief commentary on a recent *ACS Central Science* article that evaluated the performance of different laboratories in elemental analysis and suggests that a broader conclusion should be drawn instead, recognizing the benefits of metrology and the international quality infrastructure.

The recent article “An International Study Evaluating Elemental Analysis” in *ACS Central Science* by Melen et al.^1^ provided an interesting statistical viewpoint of the performance of 18 independent service providers of elemental analysis. The results obtained showed that a significant proportion of results were in excess of 0.4% from the expected values, and it was therefore proposed that imposing this limit for the deviation of results submitted to peer-reviewed journals is not a realistic requirement.

This thorough study draws an appropriate statistical conclusion from the experiment as conducted with the laboratories involved, but I believe there is a further dimension that must be explored before the full story is told. That dimension involves metrology—the science of measurement—and the global quality infrastructure, which exists to ensure and enhance the worldwide comparability of measurement. Specifically, I would contend that there is no evidence that 0.4% is not a realistic journal requirement for evaluating elemental analysis unless the competency of the analytical laboratories involved has been robustly, independently assessed. There is no description in the article of the independently assessed competency of the analytical laboratories involved. This ought to be considered in future studies of this type since, without this additional information, it is difficult to make any conclusions about what quality of analysis would be fit for its intended purpose.

Fortunately metrology^2^ and the global quality infrastructure can help us. The global quality infrastructure is “the system comprising the organizations (public and private) together with the policies, relevant legal and regulatory framework, and practices needed to support and enhance the quality, safety and environmental soundness of goods, services and processes. [...] It relies on metrology, standardization, accreditation, conformity assessment and market surveillance.”^3^ The work of this system often goes unnoticed because it is the underpinning measurement infra-technology aimed at maintaining stability, in order to make all of everyday life function: from the GPS on mobile phones, through correct doses in healthcare and drug testing in sport, to enabling complex machine parts to fit together first time and ensuring weights and measures in the local market are correct. Because metrology and the quality infrastructure are aimed at “stability, not step change,” they often do not hit the headlines, but they are essential for us to ensure the robustness of our scientific conclusions and to have confidence in the data we produce.^4^ The global quality system is already well-recognized and frequently used where measurements are made in support of regulatory requirements or within legal frameworks—for example the assessment of air pollutant levels against legal limits, or assessment of drinking water or food quality. However, there are still many other areas of science and technology that would benefit from its adoption.^5^

The activity of the quality infrastructure relevant to this discussion is the assessment of the competency of analytical laboratories, to ensure that the results they produce are stable, comparable, and traceable to the primary standards of measurement held at National Metrology Institutes (NMIs, such as NPL in the UK, or NIST in the US) and are quoted together with a measurement uncertainty that is fit for its intended purpose. Understanding measurement uncertainty is a key part of being able to draw any conclusions about whether the results produced are fit for purpose or not. Would we have more confidence in a measurement with a deviation from the expected value of (+0.20 ± 0.04)% or one with a deviation of (+0.20 ± 0.40)%? (Equally, if the latter measurement is really considered fit for purpose for our needs we perhaps need not worry as much about the quality of our analysis.) Therefore, a key requirement to ensure confidence in data is for analytical laboratories to be independently accredited by a National Accreditation Body [NAB, such as UKAS in the UK, or NVLAP (among others) in the US] to the ISO/IEC 17025:2017 documentary standard “General requirements for the competence of testing and calibration laboratories”.^6^ This demonstrates a laboratory’s ability to produce consistently valid results within a stated measurement uncertainty. Such an accreditation provides confidence that these organizations are competent and can be trusted to deliver promised levels of performance. It thereby drives confidence in all sectors by underpinning quality of results, and ensuring their traceability, comparability, and validity.

An important part of assuring the quality and comparability of measurement is regular participation in a proficiency testing (PT) scheme or interlaboratory comparison, of a similar type to that described by Melen et al.^1^ Few participants see the value in PT schemes before they take part in one, but they almost all do afterwards! Independent assessment of one’s abilities, for better or worse, is usually illuminating and always helps to improve comparability in measurement. The reference values in such a scheme would be independently assigned by an NMI based on material whose purity has been rigorously characterized. This is important since, in the case of elemental analysis, it is essential to know not only the purity of the compound precisely but also the identity and concentrations of the impurities. This is the sort of information provided by “certified reference materials”^7^ which can be used to validate analytical methods and also provide reference values for interlaboratory comparisons. As an example, NIST’s Standard Reference Material program provides a huge range of such materials for many different applications.^8^ (This reference material need not exactly match the compound of interest itself: if you can demonstrate a fit-for-purpose measurement on a relevant reference material, then your method is under control and your outcomes are quality assured.)

Therefore, I would suggest that a more appropriate, broader conclusion would be that elemental analysis for publication in peer-reviewed journals must be conducted by laboratories with a recognized level of competence, independently accredited to ISO 17025 for the measurements in question. Requiring accreditation for these analyses will give the academic community more confidence in the results they receive from third party laboratories, and will improve the quality of analysis and comparability of measurement within the community as a whole. Accreditation clearly adds costs to performing analyses, but these can be offset somewhat by having centralized analytical facilities that have high throughputs. Furthermore, the additional confidence in the conclusions provided by accredited analysis is also an (often invisible) benefit. However, ensuring that laboratories or countries with less funding do not get excluded requires ongoing attention.

Only when the quality of measurement from the analytical laboratory is properly known can the academic community genuinely judge the state-of-the-art in elemental analysis and therefore set a suitable threshold of accuracy based on the performance of independently accredited analytical laboratories. In short, 0.4% may or may not be a sensible threshold, but we won’t know that until the competence of the set of laboratories performing the measurements is independently validated.
